# Using an electronic medical record (EMR) to conduct clinical trials: Salford Lung Study feasibility

**DOI:** 10.1186/s12911-015-0132-z

**Published:** 2015-02-07

**Authors:** Hanaa F Elkhenini, Kourtney J Davis, Norman D Stein, John P New, Mark R Delderfield, Martin Gibson, Jorgen Vestbo, Ashley Woodcock, Nawar Diar Bakerly

**Affiliations:** Manchester Academic Health Science Centre, University of Manchester, Manchester, UK; NorthWest EHealth, Salford Royal NHS Foundation Trust, Salford, UK; GlaxoSmithKline R&D, Worldwide Epidemiology, Wavre, Belgium; GlaxoSmithKline R&D, Worldwide Epidemiology, Uxbridge, UK; University Hospital South Manchester, Manchester, UK

**Keywords:** Asthma, COPD, Electronic medical record, EMR, Real-world data, Primary and secondary healthcare, Dual verification

## Abstract

**Background:**

Real-world data on the benefit/risk profile of medicines is needed, particularly in patients who are ineligible for randomised controlled trials conducted for registration purposes. This paper describes the methodology and source data verification which enables the conduct of pre-licensing clinical trials of COPD and asthma in the community using the electronic medical record (EMR), NorthWest EHealth linked database (NWEH-LDB) and alert systems.

**Methods:**

Dual verification of extracts into NWEH-LDB was performed using two independent data sources (Salford Integrated Record [SIR] and Apollo database) from one primary care practice in Salford (N = 3504). A feasibility study was conducted to test the reliability of the NWEH-LDB to support longitudinal data analysis and pragmatic clinical trials in asthma and COPD. This involved a retrospective extraction of data from all registered practices in Salford to identify a cohort of patients with a diagnosis of asthma (aged ≥18) and/or COPD (aged ≥40) and ≥2 prescriptions for inhaled bronchodilators during 2008. Health care resource utilisation (HRU) outcomes during 2009 were assessed. Exacerbations were defined as: prescription for oral corticosteroids (OCS) in asthma and prescription of OCS or antibiotics in COPD; and/or hospitalisation for a respiratory cause.

**Results:**

Dual verification demonstrated consistency between SIR and Apollo data sources: 3453 (98.6%) patients were common to both systems; 99.9% of prescription records were matched and of 29,830 diagnosis records, one record was missing from Apollo and 272 (0.9%) from SIR. Identified COPD patients were also highly concordant (Kappa coefficient = 0.98).

A total of 7981 asthma patients and 4478 COPD patients were identified within the NWEH-LDB. Cohort analyses enumerated the most commonly prescribed respiratory medication classes to be: inhaled corticosteroids (ICS) (42%) and ICS plus long-acting β_2_-agonist (LABA) (40%) in asthma; ICS plus LABA (55%) and long-acting muscarinic antagonists (36%) in COPD. During 2009 HRU was greater in the COPD versus asthma cohorts, and exacerbation rates in 2009 were higher in patients who had ≥2 exacerbations versus ≤1 exacerbation in 2008 for both asthma (137.5 vs. 20.3 per 100 person-years, respectively) and COPD (144.6 vs. 41.0, respectively).

**Conclusion:**

Apollo and SIR data extracts into NWEH-LDB showed a high level of concordance for asthma and COPD patients. Longitudinal data analysis characterized the COPD and asthma populations in Salford including medications prescribed and health care utilisation outcomes suitable for clinical trial planning.

## Background

Large computerised patient databases provide a useful source of real life observational data, and the General Practice Research Database (GPRD) has been successfully used to generate descriptive epidemiology data in chronic conditions such as Chronic Obstructive Pulmonary Disease (COPD) [1-3] and asthma [1,4,5] from a large group of UK primary care practices. Historically the limitations of the GPRD for clinical research were a time gap between GP data capture and availability for the researcher and limited links to other healthcare databases, although these are currently being addressed with the development of the Clinical Practice Research Datalink (CPRD) and in ongoing pilot work for Phase 4 pragmatic clinical trials [6,7]. The use of electronic medical record (EMR) data in health research is a key objective in the Department of Health’s national research strategy [8]. EMR is increasingly adopted to support both efficiency and quality of patient care and to facilitate clinical research. Several studies have described the design and implementation of EMR, electronic data capture (EDC), data extraction and EMR retrieval systems to enable accurate and efficient data entry for clinical research to be performed on-site in real time [9-11].

Asthma and COPD are both treatable diseases that can be well managed [12,13], but despite this, a large proportion of asthmatics are poorly controlled [12,14,15], and COPD remains under-diagnosed and under-treated [2,16]. In everyday clinical practice, variations in asthma control and prescribing patterns across countries have been reported, as well as differences in disease perceptions amongst physicians and patients [17-19]. Similarly for COPD, variations in treatments, standards of care and adherence to guidelines have been reported across different geographical regions [20-23]. In asthma and COPD, the application of EMR retrieval systems would enable the monitoring of large patient populations to support evaluation of comparative effectiveness, safety, and health care resource utilisation (HRU) of treatments in a real life setting.

This paper describes the methodology of development of the NorthWest EHealth linked database (NWEH-LDB) and alert systems. The main outcome of the study was an assessment of the reliability of NWEH-LDB as a platform that can be used to support the delivery of pragmatic clinical trials. A retrospective analysis of asthma and COPD cohorts, identified within the database, was conducted as part of a feasibility assessment to evaluate whether this system could provide a feasible and valid platform for conducting pragmatic clinical trials.

## Methods

### North West EHealth linked database (NWEH-LDB) and alert systems

**Figure 1:**
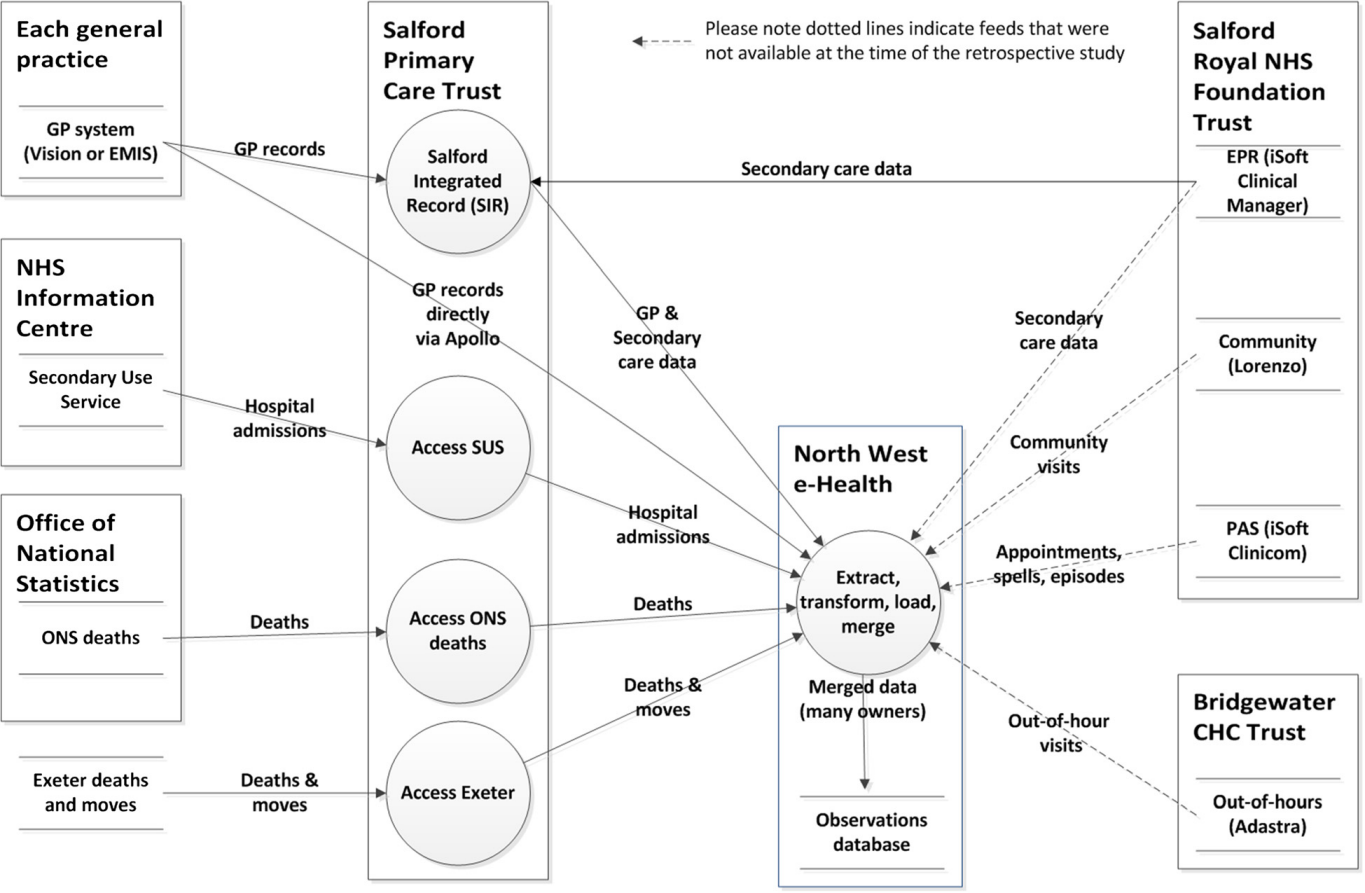
**Schematic of North West EHealth database and alert systems.**

NWEH-LDB and alert systems were designed to provide a comprehensive, daily updated, electronic patient-level database including a range of data sources (Figure 1). It links primary and secondary care data with 24-hour download intervals to a secure server to provide an integrated EMR linked by the patient’s NHS number. The system enables the extraction of electronic data from primary care practices in Salford (EMIS and VISION); Salford Royal NHS Foundation Trust (iSOFT and Allscripts); out of hours’ service (Secondary Uses Service (SUS) [24]), Salford integrated record (SIR) and Hospital Episode Statistics (HES) [25]. A third party information technology system (Apollo) which is used by primary care practices to monitor their own performance and regulatory compliance was used in our study to cross validate SIR. Apollo medical systems’ software integrates with GP clinical systems to extract the required data, which is then presented in an anonymous, encrypted and standardised format. Both SIR and Apollo systems are run by independent teams of IT specialists via different software and hardware. Tools to extract, transform and load data are used to combine feeds from all the previously mentioned sources in a single database (referred to as NWEH-LDB) (Figure 1).

SIR holds electronic records for approximately 300,000 patients registered with 53 primary care practices in Salford [26]. It was set up in 2004 in Salford, UK to share patients’ information electronically between local healthcare providers including secondary and primary care. Its main purpose is to facilitate clinical care of patients with long term conditions and to support the Quality of Care Framework [27]. Patients can choose to opt out of SIR or part of it at any time. SIR data consists of two files: the patient file – a list of (anonymised) patient identifiers together with age and sex, and a journal file consisting of all the GP records for each patient in the practice, labelled by patient identifier. The journal file was split into *prescription data*, a list of all the prescriptions for each patient and *event data*, all the remaining data such as diagnoses, lab test results and blood pressure measurements. The Apollo data are similarly structured. In comparison to SIR, the NWEH-LDB provides a comprehensive and daily updated electronic patient record and has the potential to alert end users (healthcare providers or researchers) of the occurrence of selected events according to any predefined criteria. This function aids the remote monitoring of patients’ events (HCU or safety events) since every time a patient is in contact with a health care professional this leaves an electronic footprint (e.g. note entry, prescription or blood results) which is subsequently picked up by NWEH-LDB. Events that satisfy predefined parameters are displayed as electronic alerts on the activity summary of NWEH-LDB. Daily update of the activity summary is accessible to the pre-specified/qualified end users for review and investigation of possible safety adverse events.

### Dual verification of data sources

The main objective of the study was to assess the reliability of NWEH-LDB. Anonymised patient data from one large primary care practice in Salford (N = 3504 registered adults) were extracted using two independent sources: SIR and Apollo. Although multiple data sources were used for the creation of the NWEH-LDB, SIR and Apollo are the most important sources of direct patient care information and were therefore considered the most likely to result in potential data anomalies. In addition, it was not feasible to cross-verify other national data sources such as SUS [24] and HES [25].

Salford general practices with IT facilities that run overnight and during weekends to permit data extraction were approached. EMR data were obtained from general practices during the period between 1st February 2010 to 1st November 2011. A patient matching algorithm was devised to correlate each patient record assigned by SIR with Apollo’s patient record. Attention was then restricted to the matched patients and the number of patients with a diagnosis of COPD to evaluate any discordance. Finally the Apollo and SIR prescription records were merged. Duplicates were removed, thereby leaving only records unique to either SIR or Apollo.

The aims of the process were three-fold: a) establish that the SIR extract contained data on all patients; b) show that the identification of the asthma and COPD cohorts used for the retrospective analysis were correctly attributed and c) demonstrate that all the prescription and event data for each patient had been captured.

### Retrospective cohort analysis using the NWEH-LDB

As part of assessing the feasibility of future pragmatic trial utilizing NWEH-LDB, a retrospective cohort study to identify anonymised patients with a coded diagnosis of either asthma or COPD in Salford using compatible Read Codes [28] during 2008 with follow up through 2009 was conducted, using data from all registered practices in Salford. Eligible patients were required to: (1) have had a GP diagnosis of asthma or COPD during or before 2008; (2) have been prescribed at least two prescriptions for any short- or long acting- bronchodilator medication between October 2007 and December 2008, in order to ensure patients had evidence of current disease activity; and (3) be aged ≥18 years (asthma) or ≥40 years (COPD). Patients were excluded if they had recorded primary diagnoses of cystic fibrosis, lung cancer, bronchiectasis or fibrotic lung disease anytime in 2008–2009. In addition, patients were excluded from the asthma cohort if they had evidence of either severe asthma (>90 days’ supply of OCS during 2008) or a co-diagnosis of COPD. Patients in the COPD cohort were permitted to have a co-diagnosis of asthma. About 80% of COPD patients had spirometry measurements recorded confirming the diagnosis.

The retrospective cohort study was approved by the independent NorthWest EHealth research ethics board and the SIR ethics board.

#### Feasibility study

The identified asthma and COPD cohorts were analysed to assess the feasibility of conducting a future clinical trial design. Data from the baseline year (2008) were used to establish patient eligibility and demographic and clinical characteristics, including lung function data which were based on the first measurements recorded (and coded) by the GP.

Outcome measures were rates of asthma and COPD moderate/severe exacerbations and health resource utilisations (HRU) during 2009. Asthma exacerbations were defined as a prescription of OCS and/or hospitalisation for a respiratory cause. COPD exacerbations were defined as a prescription of antibiotics and/or OCS and/or hospitalisation for a respiratory cause. HRU outcomes were assessed by prescriptions of short courses of OCS and/or antibiotics; overall number of GP visits (routine and unscheduled appointments); number of hospital admissions (overall and respiratory-specific) and number of days spent in hospital.

#### Statistical analysis

A concordance analysis (Kappa) was used to quantify the association between the number of patients with COPD identified by SIR and Apollo [29]. A Kappa coefficient was calculated whereby a value of 1 indicates a perfect agreement.

As the feasibility analysis was descriptive in nature, no power calculations were performed. All patients listed on the NWEH-LDB with a coded diagnosis of asthma or COPD, and who met the eligibility criteria, were included in the analysis. Descriptive statistics were generated for each cohort (asthma; COPD; COPD subset with co-morbid asthma) using Microsoft SQL server 2008 and Stata™ programs [30]. The frequency counts and percentages of patients with events of interest were calculated for the baseline period (2008). The frequency counts and rates of events per person-years of follow-up were calculated for the 2009 data, using all the months of follow-up available in the record. Cohort exit date was defined as date of death, exit from the NorthWest EHealth register due to notice of registration in another region, or December 31 2009, whichever came first.

## Results

### Dual verification of data sources

**Figure 2:**
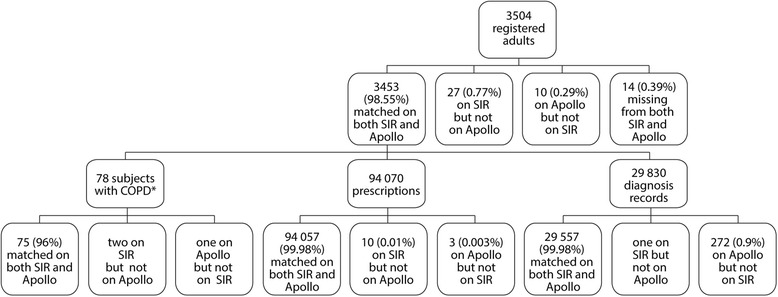
**Concordance of COPD patient numbers, prescription records and diagnosis records between SIR and Apollo systems.**

The matching algorithm was able to identify 3446 corresponding registered patients electronically and a further 7 patients manually, leaving a remainder of 27 patients unique to SIR and 10 unique to Apollo (Figure 2). The number of patients that both Apollo and SIR failed to detect is unknown, but is likely to be small. According to the Exeter database (record of all patients registered with a NHS GP in England and Wales), there were a total of 3504 patients aged 18 or over registered in the second quarter of 2010–11 [31]. Part of any discrepancy was explained by some patients refusing consent for their records to be held in electronic form, but resulted in very few patients being omitted from the SIR extract. With respect to the patients with a diagnosis of COPD, there were 76 such patients according to Apollo and 77 according to SIR, with 75 being common to both (kappa = 0.98) (Figure 2).

Following the merging of 94,070 prescription records from the two datasets, the majority (99.98%) of data were matched on the SIR and Apollo systems (Figure 2). A total of 3 records were missing from SIR and 10 from Apollo.

Out of 29,830 diagnosis records, one diagnosis record was missing from Apollo and 272 (0.9%) from SIR.

### Retrospective cohort analysis using the NWEH-LDB

#### Description of asthma and COPD populations in Salford

**Figure 3:**
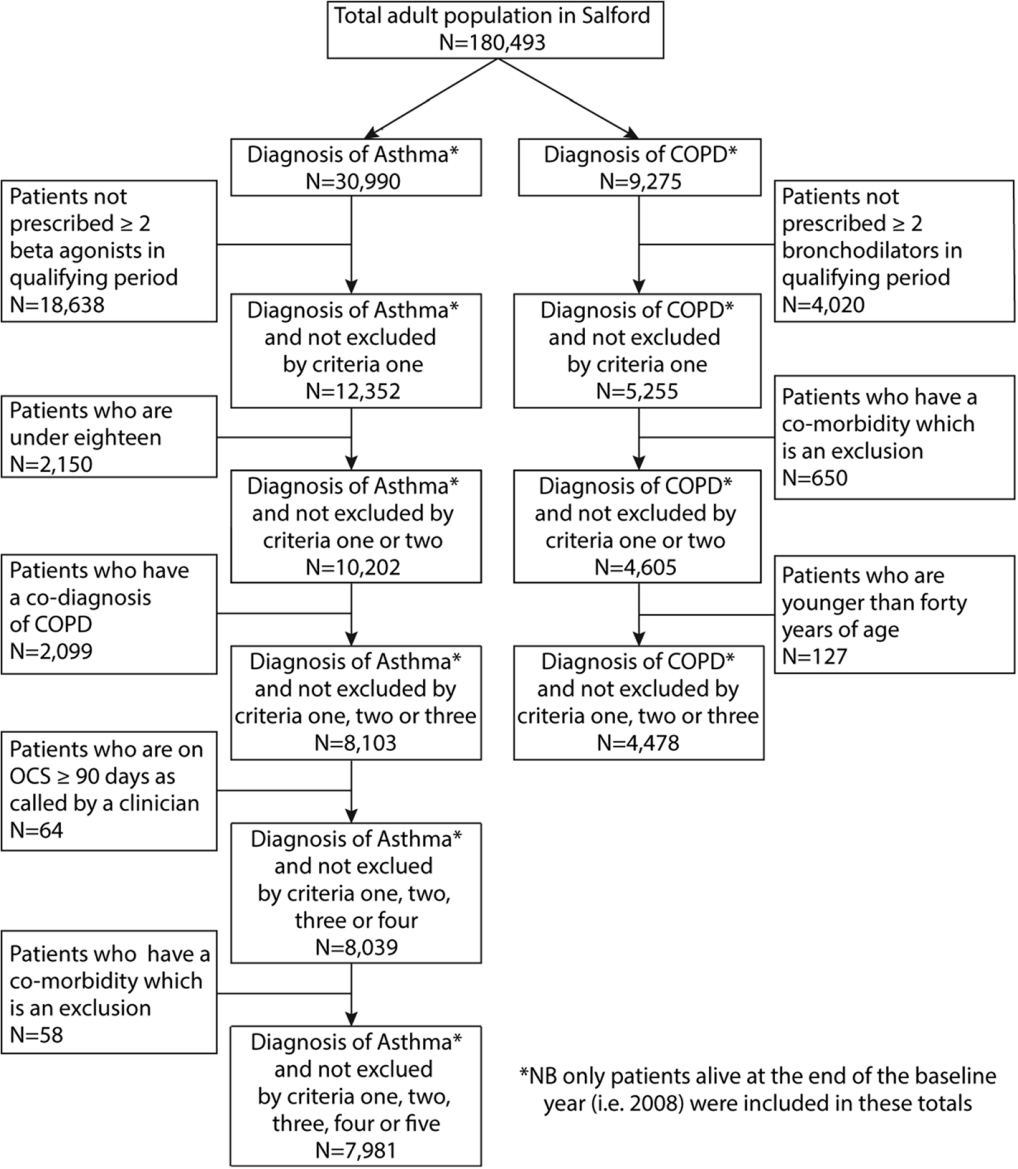
**Flow of patients in the retrospective cohort study.**

A population of 180,493 adults aged ≥18 years were identified in the NWEH-LDB, of which 90,706 were male. The schematic flow of patients included/excluded in the study for both the asthma and COPD cohorts is shown in Figure 3. Of the total adult population ages 18 years and older, 7981 had a coded diagnosis of asthma (4.4%) and 4478 had a coded diagnosis of COPD (2.5%). Within the COPD cohort, 1718 patients also had a diagnosis of asthma. Asthma prevalence was higher in females (5.3%) than males (3.5%); COPD prevalence was similar in both sexes (females: 2.7%; males: 2.2%).

A summary of baseline and clinical characteristics by disease cohort is presented in Table 1. Asthma patients had a mean age of 33 years and FEV_1_ (percent predicted) of 2.42 L (86%). More than 82% of asthma patients were prescribed inhaled corticosteroids (ICS), of which 40% were ICS plus long-acting β_2_-agonist (ICS/LABA) combination inhalers, and 42% were ICS monotherapy. The proportion with co-morbidities was low, except for hypertension (25%).

**Table 1:** **Baseline and clinical characteristics of asthma and COPD cohorts (2008 data)**

	**Asthma only cohort**	**COPD cohort**	**COPD and asthma cohort** ^**1**^
	**(N = 7981)**	**(N = 4478)**	**(N = 1718)**
Age at diagnosis (years), mean (SD)	33.1 (20.0)	61.1 (12.9)	59.2 (13.6)
Male sex, n (%)	3209 (40)	2026 (45)	672 (39)
Smoking history, n (%)			
Current	2148 (27)	1796 (40)	639 (37)
Former	2579 (32)	2326 (52)	855 (50)
Never	3241 (41)	356 (8)	224 (13)
Body Mass Index, n (%)			
<18	88 (1)	160 (4)	41 (2)
18-25	2370 (30)	1686 (38)	601 (35)
26-30	1772 (22)	1064 (24)	407 (24)
>30	2310 (29)	1161 (26)	510 (30)
unknown	1441 (18)	407 (9)	159 (9)
FEV_1_ (L), mean (SD)	2.42 (0.83)	1.45 (0.60)	1.48 (0.63)
FEV_1_% predicted, mean (SD)	86.0 (18.4)	61.0 (19.7)	62.7 (20.0)
GOLD Stage, n (%)			
I	n/a	583 (13)	247 (14)
II	n/a	2036 (45)	713 (42)
III	n/a	874 (20)	307 (18)
IV	n/a	200 (4)	57 (3)
unknown	n/a	785 (18)	394 (23)
Peak expiratory flow, mean (SD)	393.7 (123.8)	n/a	n/a
Medication, n (%)			
SABA	7492 (94)	4189 (94)	1638 (95)
LABA	777 (10)	522 (12)	252 (15)
LAMA	49 (<1)	1617 (36)	515 (30)
LTRA	359 (4)	119 (3)	91 (5)
ICS monotherapy	3364 (42)	652 (15)	332 (19)
ICS plus LABA^2^	3190 (40)	2484 (55)	1128 (66)
ICS plus LTRA or LAMA	52 (<1)	153 (3)	53 (3)
Cardiovascular comorbidities,			
n(%)^3^			
Any	2176 (27)	2586 (58)	964 (56)
Acute MI	226 (3)	492 (11)	150 (9)
Hypertension	2016 (25)	2224 (50)	852 (50)
Stroke	89 (1)	226 (5)	68 (4)
Heart failure	136 (2)	467 (10)	166 (10)

^1^Subset of COPD cohort; ^2^taken in combination or as separate inhalers; n/a = not applicable; ^3^present before 31 December 2009.

FEV_1_: forced expiratory volume in 1 sec; GOLD: Global Initiative for Chronic Obstructive Lung disease; SABA: short-acting β_2_-agonist; LABA: long-acting β_2_-agonist; LAMA: long-acting muscarinic antagonist; LTRA: leukotriene receptor antagonist; ICS: inhaled corticosteroid; MI: myocardial infarction.

Compared to the asthma cohort, COPD patients were older, in keeping with the criterion of excluding those younger than age 40 and the disease aetiology (mean 61 years) with lower mean FEV_1_, 1.45 L (61%). Just under half (45%) were classified as GOLD Stage 2, according to the lung function data. Over half of patients (55%) were prescribed an ICS/LABA combination inhaler, 36% a long-acting muscarinic antagonist (LAMA), and 15% ICS monotherapy. Levels of cardiovascular co-morbidity were high; 50% of patients had comorbid diagnosis of high blood pressure and 15% had suffered a myocardial infarction or stroke.

Approximately 14% of the patients had a label of both COPD and asthma in the EMR. The subset of COPD with comorbid asthma had almost identical characteristics to the COPD alone population, in terms of smoking history, FEV_1_ and prescribed medication.

#### Health resource utilisation

In 2009, OCS and antibiotics prescriptions, GP visits, and hospitalisations per 100 person-years were greater for patients defined as having COPD or COPD/asthma compared with those with asthma only (Table 2). Prescription rates of OCS were 3 times higher and antibiotic prescription rates were 1.5 times higher for COPD than for asthma. All-cause hospitalisation and respiratory admissions were approximately 2- and 5- fold higher, respectively, for COPD compared to asthma which was consistent with the older age and higher morbidity profile in COPD.

**Table 2:** **12 month resource utilisation data per 100 person-years during 2009 (subsets predetermined from data collected during 2008)**

	**Asthma only**				**COPD** ^**1**^				**COPD and asthma cohort** ^**1**^			
	**Total**	**Subset ≤1 EXAC**	**Subset 2 EXAC**	**Subset ICS or ICS/LABA**	**Total**	**Subset ≤1 EXAC**	**Subset 2 EXAC**	**Subset ICS/LABA LABA or LAMA**	**Total**	**Subset ≤1 EXAC**	**Subset 2 EXAC**	**Subset ICS/LABALABA or LAMA**
N	7981	7525	456	6606	4478	2247	2231	2997	1718	812	906	1223
Short courses oral steroids^2^	26.9	20.3	137.5	29.8	92.5	41.0	144.6	118.6	103.9	44.6	157.4	127.5
Antibiotics	96.8	90.7	197.6	100.0	180.6	100.9	261.2	206.6	196.4	108.4	275.3	218.5
GP visits - all cause^3^	1068.2	1041.9	1515.7	1097.2	1525.9	1296.5	1757.9	1599.6	1531.1	1285.9	1751.0	1587.0
Hospitalisations - all cause	25.5	25.3	29.6	23.3	53.4	51.3	55.4	53.3	46.2	37.1	54.3	50.6
Hospitalisations - respiratory	2.8	2.6	6.0	2.9	13.6	9.1	18.0	16.6	14.3	10.5	17.7	17.5

^1^Subset of COPD cohort; ^2^Data for patients who received >12 OCS prescriptions during 2008 were excluded as outliers; ^3^routine and unscheduled.

EXAC: asthma or COPD exacerbation; GP: general practitioner; ICS: Inhaled corticosteroid; LABA: long-acting β_2_-agonist; LAMA: long-acting muscarinic antagonist.

**Figure 4:**
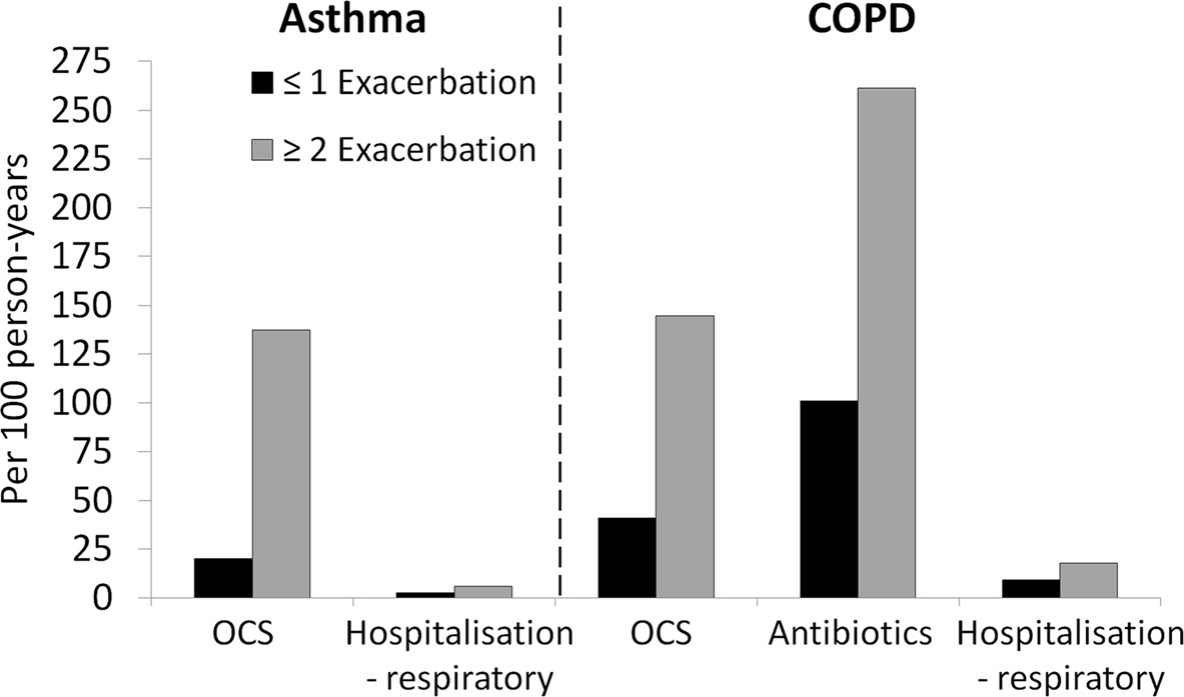
**Exacerbation rate during 2009, stratified by infrequent (≤1) or frequent (≥2) exacerbation status in 2008, for asthma and COPD cohorts.**

Resource utilisation for both asthma and COPD in 2009 was greater for a subset of patients who had evidence of ≥2 exacerbations in 2008 compared with either the total asthma and COPD cohorts respectively, or a subset of patients who had evidence of ≤1 exacerbation (Table 2; Figure 4). This pattern was observed for all types of resource use: prescriptions of OCS and antibiotics, number of GP visits and number of hospitalisations.

The average number of days spent in hospital per patient per year for the asthma cohort was 1.8 days, 12% of which were for respiratory reasons. Patients in the COPD cohort spent an average of 6.7 days in hospital of which one-third were due to respiratory reasons (data not shown).

## Discussion

Results from these analyses suggest that the NWEH-LDB is able to provide the necessary elements of a platform for real-world benefit/risk assessment of clinical studies. NWEH-LDB enabled the identification of asthma and COPD cohorts and data extraction on prescriptions and healthcare events for all patients. We can speculate that these data include sufficient detail to produce alerts for patient safety and HRU monitoring during clinical trials. The dual verification exercise confirmed high concordance between the two independent data sources, SIR and Apollo. Only 37 data records from 3504 registered patients were discordant between the systems. Most of the discrepancies appear to be due to patients who have either transferred to another practice or died. Good concordance was also demonstrated between SIR and Apollo for prescription and events records. By collating data from multiple sources, NWEH-LDB is sufficiently robust to compensate for the small percentage of diagnosis records (0.9%) missing from the SIR database (Figure 1).

Previous studies demonstrate various methods to deploy EMR in clinical research. For instance, Goodman and colleagues explored solutions to integrate the EMR and EDC systems to enable accurate and efficient collection of cancer clinical trial data [9]. Murphy et al. [11] described the implementation of an EMR and data extraction techniques to facilitate real time and on-site data entry for clinical research. They used special ‘study management’ screens to capture additional data needed for clinical trials such as adverse events, enrolment, termination and missed visits. Similarly, Yamamoto et al. developed an EMR retrieval system to identify patients who met clinical research eligibility criteria [10]. Clinical trial alert systems are also implemented in order to increase physician participation in clinical trials [32] and improve recruitment [33,34]. In line with these studies, we introduce a template to collate electronic patient data from readily available systems in hospital and general practice with the capability to extract information as required for research. Similar to Murphy et al. [11], the NWEH-LDB systems use real time and on-site data. Our systems provide a generic platform that can extract data previously defined according to the researchers’ interest. Here we describe an example of retrospective data extraction and analysis in asthma and COPD to demonstrate the reliability of this new template in health utilisation and outcome research.

Our systems are not intended to support recruitment. However, the retrospective cohort study identified patients with asthma and/or COPD, based on primary or secondary care diagnoses, together with their prescribing data, and provided longitudinal data on HRU and clinical endpoints of interest. These data were utilized in assessing feasibility of a future trial when compared to specific protocol elements, including endpoint selection and sample size calculation [35]. Data extraction process via NWEH-LDB can run on a daily basis with a potential to create alerts each time patients have contact with healthcare providers, prescriptions or laboratory results. These functions could be applied in pragmatic clinical trials to support patient safety monitoring remotely and obtain real life evidence in the future.

Comparisons of our data for the asthma and COPD cohorts with other population based studies [2,4,36] put our findings in context and provide additional evidence that the NWEH-LDB provides accurate and robust data to characterize disease severity, treatment, and outcomes. With respect to the findings on history of exacerbations, the results for the asthma cohort concur with the recent review by Dougherty and Fahy, which stated that a history of one or more exacerbation is an important risk factor for recurrent exacerbations suggesting an “exacerbation-prone” subset of asthmatics [37]. There is also increasing evidence that different phenotypes exist in COPD [38,39] and the evidence supports the existence of a COPD frequent-exacerbator phenotype [40,41]. A recent retrospective analysis of the ECLIPSE cohort data showed that, although the frequency and severity of exacerbations were more severe as COPD progressed, the single best predictor of exacerbations was having a history of exacerbations [40]. The NWEH-LDB data also provide evidence of a higher rate of COPD exacerbations in 2009 among those patients who had two or more moderate/severe exacerbations recorded in 2008.

Some of the limitations of electronic medical record data reflect the environment and purpose of its collection, namely they are not recorded systematically under strictly controlled conditions, as in traditional bespoke clinical research, and rely on the GP and other contributors to accurately complete the records for all patients equally. In addition, not all GP’s routinely participate in research and those participating in a future EMR-based clinical study may be more diligent about recording information well compared with GPs not participating in research. Another limitation of this dual verification process pilot study is that the data were generated from one GP practice, which was not randomly selected, and therefore may not be representative of the whole group of participating practices included in the retrospective cohort study.

Data from this study have several noteworthy implications. Firstly, retrospective EMR data give a real-life picture of the management of COPD and asthma patients in an unperturbed clinical setting, without the constraints of strict randomised controlled trial criteria. Secondly, multi-source databases like NWEH-LDB can be used to monitor changes in a cohort of interest (in this case Asthma and COPD) over a predefined time period if data are extracted from sources into NWEH-LDB at regular intervals. For example, changes in the trends of disease prevalence and comorbidities would require data extraction from sources over long time periods (i.e. years); however, the monitoring of prescribing trends, HRU, or admissions would require narrow extraction intervals depending upon the research question. Therefore, NWEH-LDB model has the potential to provide data necessary to inform the study design, planning and power calculations of real-life safety and effectiveness studies. Since the feasibility study was conducted, NWEH-LDB has been further developed with additional resources and links incorporated (Figure 1), increasing the capability for conducting successful future EMR-enabled trials.

While linked EMR platforms continue to improve in the quantity and quality of data linkages, there may be a need for flexibility in the data collection process to augment EMR for pre-defined efficacy, effectiveness, or safety events of interest to meet requirements for certain RCTs. For example, exacerbations of COPD may require an electronic case report form (eCRF) or link to a patient portal for recording a validated Patient Reported Outcome (PRO) to be added to the primary care software to capture the indication for OCS/antibiotic prescription or measure asthma control, as relying on the e-prescription data alone may not be specific enough and could lead to misclassification. Furthermore, an ideal system must be sensitive enough to capture all major safety events during a trial, with the potential for blinded adjudication and rapid reporting/follow-up.

## Conclusions

Data collected via NWEH-LDB using sources from SIR and Apollo showed high levels of concordance and provided detailed information on the COPD and asthma population including details on HRU in addition to prescribing data and clinical end-points of interest.

These features have the potential to enable real-world data collection by a research network of general practitioners using EMR with flexible eCRF modifications and link to pharmacy dispensing to provide a platform for assessing novel versus standard treatments in phase 3 or 4 clinical trials. Data from these types of studies could provide complementary information in a more representative and heterogeneous group of patients than traditional RCTs, during the regulatory approval process, with the added value of demonstrating the benefit/risk profile of a drug compared to standard care treatments in the usual care environment. This type of study may be able to enrol a wider range of target patients more efficiently than traditional RCTs by recruiting at their usual source of care, ideally speeding the time to meaningful evaluations as part of a learning healthcare system with public health impact of improved decision making.
